# Aurora kinase A (AURKA) interaction with Wnt and Ras-MAPK signalling pathways in colorectal cancer

**DOI:** 10.1038/s41598-018-24982-z

**Published:** 2018-05-14

**Authors:** Annika Jacobsen, Linda J. W. Bosch, Sanne R. Martens-de Kemp, Beatriz Carvalho, Anke H. Sillars-Hardebol, Richard J. Dobson, Emanuele de Rinaldis, Gerrit A. Meijer, Sanne Abeln, Jaap Heringa, Remond J. A. Fijneman, K. Anton Feenstra

**Affiliations:** 1 Centre for Integrative Bioinformatics (IBIVU), Vrije Universiteit Amsterdam, Amsterdam, The Netherlands; 2 Department of Pathology, The Netherlands Cancer Institute, Amsterdam, The Netherlands; 3 Department of Pathology, VU University Medical Center, Amsterdam, The Netherlands; 4 Bioinformatics group at the NIHR Biomedical Research Centre for Mental Health (IOP) and the South London and Maudsley NHS Trust, London, UK; 5 The BRC Translational Bioinformatics Unit at the NIHR Biomedical Research Centre at Guy’s and St Thomas’ NHS Foundation Trust and King’s College London, London, UK

## Abstract

Hyperactivation of Wnt and Ras-MAPK signalling are common events in development of colorectal adenomas. Further progression from adenoma-to-carcinoma is frequently associated with 20q gain and overexpression of Aurora kinase A (AURKA). Interestingly, AURKA has been shown to further enhance Wnt and Ras-MAPK signalling. However, the molecular details of these interactions in driving colorectal carcinogenesis remain poorly understood. Here we first performed differential expression analysis (DEA) of *AURKA* knockdown in two colorectal cancer (CRC) cell lines with 20q gain and AURKA overexpression. Next, using an exact algorithm, Heinz, we computed the largest connected protein-protein interaction (PPI) network module of significantly deregulated genes in the two CRC cell lines. The DEA and the Heinz analyses suggest 20 Wnt and Ras-MAPK signalling genes being deregulated by AURKA, whereof *β-catenin* and *KRAS* occurred in both cell lines. Finally, shortest path analysis over the PPI network revealed eight ‘connecting genes’ between *AURKA* and these Wnt and Ras-MAPK signalling genes, of which *UBE2D1*, *DICER1*, *CDK6* and *RACGAP1* occurred in both cell lines. This study, first, confirms that AURKA influences deregulation of Wnt and Ras-MAPK signalling genes, and second, suggests mechanisms in CRC cell lines describing these interactions.

## Introduction

Colorectal cancer (CRC) is the third most common cancer in men and the second most common cancer in women worldwide^1^. In the early stages of CRC development, proliferative signalling is sustained by hyperactivation of the Wnt and Ras-MAPK signalling pathways due to mutations in key regulatory genes^2^. Disruption of Wnt signalling, caused by mutations in the *APC* tumour suppressor gene or other genes such as *CTNNB1* (hereafter referred to as *β-catenin*), *AXIN1* or *AXIN2*, promotes the progression from normal colon epithelium to a benign precursor lesion, called adenoma^3^. Subsequently, adenoma-to-carcinoma progression is driven by further genetic and epigenetic alterations. For example, in addition to the Ras-MAPK pathway, activated by mutation in e.g. the *KRAS* gene, other pathways important in carcinoma development are the TGFβ pathway, disrupted by mutation in e.g. *SMAD4*, and the TP53 pathway, disrupted by mutations in the *TP53* gene^4,5^. Recently, it has been shown that adenoma organoids harbouring all these mutations can induce invasive cancers in mice only when a background of chromosomal instability is present^6^. This signifies the importance of chromosomal instability, which in fact occurs in ~85% of CRC^7^, and is characterized by gross chromosomal aberrations.

Chromosomal arm 20q is frequently gained in CRC^8,9^ and has a strong association with the progression of colorectal adenoma to carcinoma^10^. Aurora kinase A (AURKA), a gene coding for a key cell cycle regulator, is located on 20q. There is a significant correlation between the 20q copy number and increased AURKA mRNA and protein expression^11^. Gain of 20q and/or AURKA overexpression is associated with a poor prognosis in many cancer types including CRC^12–17^.

AURKA overexpression has been shown to stabilize β-catenin levels and thereby activating Wnt signalling in gastric cancer cells by phosphorylating the negative regulator of β-catenin, GSK3B^18,19^. Also in glioma-initiating cells (distinguished by their capacity of self-renewal) AURKA is a negative regulator of β-catenin, by binding to AXIN1^20^. Recently, it has been shown that AURKA upregulates Ras-MAPK signalling by interacting with the H-RAS/Raf-1 complex in kidney cells^21^. In addition, AURKA itself has been shown to be a target gene of both MAPK1/ERK2 signalling in pancreatic cancer cells^22^ and Wnt/β-catenin signalling in multiple myeloma^23^. These data suggest a positive feedback loop from hyperactive proliferative signalling to AURKA overexpression, further inducing proliferative signalling cells^21^.

All this implies that there is interplay between AURKA and the Wnt and Ras-MAPK signalling pathways and vice versa in different cancer settings. For Wnt and Ras-MAPK signalling, much of the mechanisms have been elucidated, also in relation with CRC^24^, but such detail is not available for the interplay with AURKA. Although different molecular mechanisms are observed in the different settings this argues that the regulation itself is important. In this study, we used two distinct cell lines, SW480 and Caco2, both derived from colon carcinomas with 20q copy number gain and mutated TP53. However, the genetics differ between these cell lines, they originate from different individuals and therefore have different germline variations, and they have progressed to carcinomas independently and therefore also differ in their somatic alterations, such as DNA mutations and DNA copy number alterations. The distinct DNA copy number profiles of the two cell lines are shown in Supplementary Fig. S1. Further, a comparative study of colon cancer cell lines showed that out of five critical cancer genes (KRAS, BRAF, PIK3CA, PTEN, and TP53) only the mutation status of KRAS differed between the two cell lines (mutated in SW480). Further, of the other four genes TP53 was mutated in both cell lines^25^. Using SW480 and Caco2, we set out to investigate which key players and molecular interactions are involved in the interplay between AURKA and the Wnt and Ras-MAPK pathways that may drive progression of CRC.

## Results

### Differentially mRNA expression analysis

**Figure 1:**
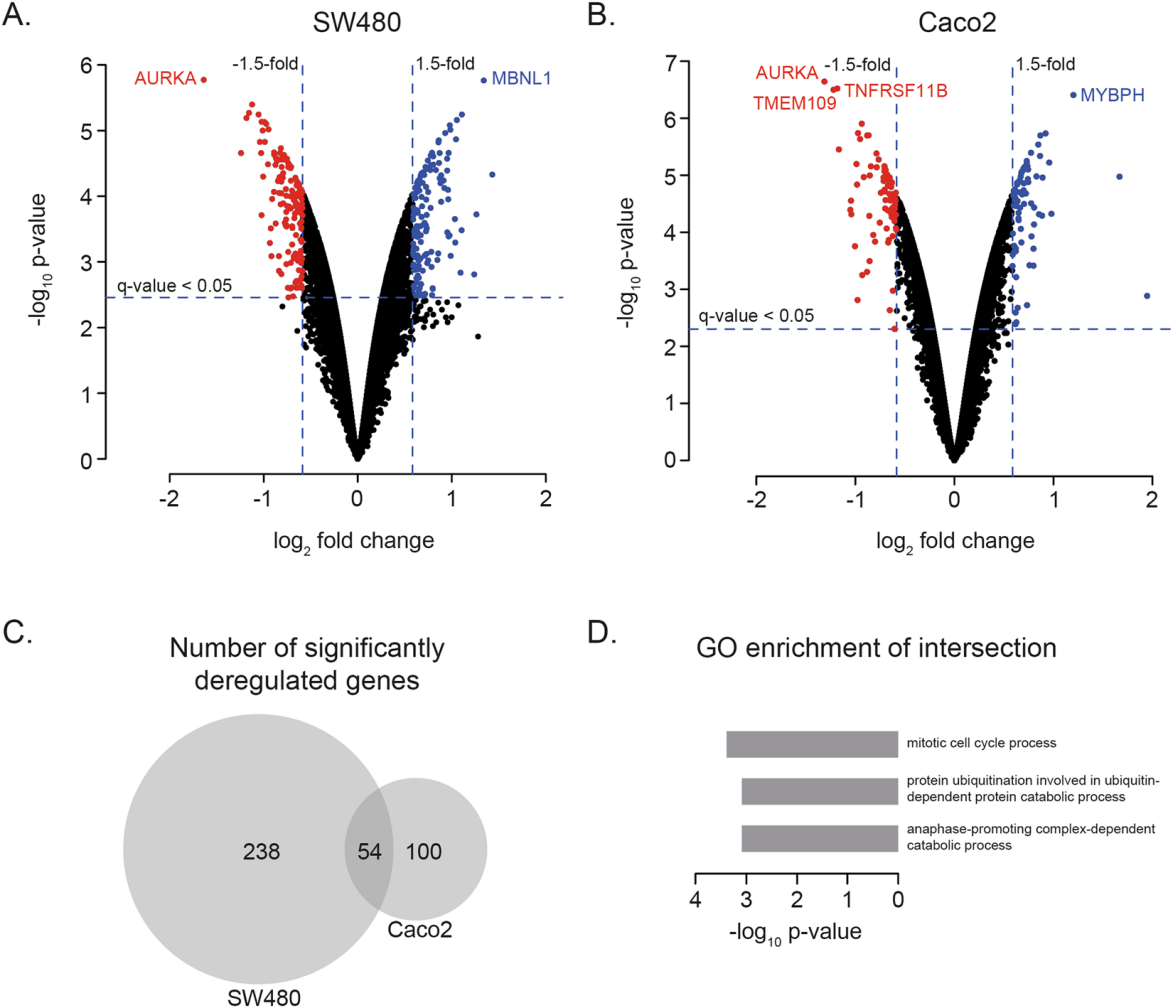
Differential mRNA expression analysis of *AURKA* knockdown in SW480 (**A**) and Caco2 (**B**) colorectal cancer cell lines. (**A**) and (**B**) Volcano plot of all unique genes from the differential mRNA expression analysis upon *AURKA* downmodulation for the SW480 and Caco2 cell lines, respectively. The vertical and horizontal lines illustrate the boundaries between significantly downregulated genes (red dots), significantly upregulated genes (blue dots) and non-significant differential genes (black dots). Horizontal lines represent the significance threshold of the q-value < 0.05. Vertical lines represent the significance threshold of the fold change 1.5 (log_2_ fold change 0.58) up or down. C) VENN diagram of the number of significantly deregulated genes in SW480 and Caco2 (see Table 1). D) GO enrichment of the genes (n = 53) that are significantly deregulated in both cell lines in the same direction (the intersection in the Venn diagram in C minus one gene).

Differential expression analysis (DEA) was performed upon *AURKA* siRNA-directed downmodulation in two CRC cell lines with 20q gain and AURKA overexpression, SW480 and Caco2. *AURKA* was the most differentially downregulated gene in both cell lines (p-value of 1.69e-6 in SW480 and 2.28e-7 in Caco2), indicating that the siRNA experiment was successful. The number of significantly expressed genes in response to *AURKA* downmodulation at a q-value less than 0.05 was 2,057 and 3,606 in SW480 and Caco2, respectively. 924 genes were significantly deregulated in both cell lines, whereof 50 were deregulated in opposite directions. In our analysis, however, we applied a more stringent threshold to determine significantly deregulated genes including both a q-value less than 0.05 and a fold change greater than 1.5 or less than −1.5. This resulted in a 292 and 154 significantly deregulated genes in SW480 and Caco2, respectively. Of the 292 genes significantly deregulated in SW480, 139 genes were upregulated and 153 downregulated (Fig. 1A and Supplementary Table S1). Of the 154 genes significantly deregulated in Caco2, 73 genes were upregulated and 81 downregulated (Fig. 1B and Supplementary Table S2). Fifty-four genes were significantly deregulated in both cell lines (Fig. 1C and Table 1). Fifty-three of these 54 were deregulated in the same direction in both cell lines: 28 up in both and 25 down in both. The only gene deregulated in different directions was *SLC12A2*, which was up in Caco2 and down in SW480. These 53 genes are interesting candidates for the general influence of AURKA. GO enrichment analysis of these genes revealed that ‘mitotic cell cycle’ was the most significantly enriched process (Fig. 1D).

**Table 1:** Genes significantly deregulated in both the Caco2 and SW480 cell lines under *AURKA* knockdown (n = 54).

Gene ID	Gene name	SW480			Caco2		
		p-value	q-value	FC**	p-value	q-value	FC**
AURKA	Aurora kinase A	1.69e-6	1.86e-2	−1.64	2.28e-7	1.96e-3	−1.31
REEP5	Receptor accessory protein 5	2.20e-5	1.86e-2	−1.24	1.83e-6	5.18e-3	−0.97
SLC12A2	Solute carrier family 12 member 2	5.70e-6	1.86e-2	−1.05	1.39e-5	6.82e-3	0.70
DICER1	Dicer 1, ribonuclease III	1.94e-4	2.35e-2	−1.02	6.79e-5	1.00e-2	−0.75
FEZ2	Fasciculation and elongation protein zeta 2	7.41e-6	1.86e-2	−0.99	7.61e-6	6.82e-3	−0.70
KRAS	KRAS proto-oncogene, GTPase	9.58e-6	1.86e-2	−0.95	1.10e-5	6.82e-3	−0.65
UBE2D1	Ubiquitin conjugating enzyme E2 D1	3.08e-4	2.55e-2	−0.92	1.08e-5	6.82e-3	−0.91
TM4SF1	Transmembrane 4 L six family member 1	1.10e-4	2.28e-2	−0.91	2.03e-5	7.98e-3	−0.74
ACVR1	Activin A receptor type 1	2.60e-4	2.46e-2	−0.87	6.36e-6	6.82e-3	−0.99
MARVELD2	MARVEL domain containing 2	2.61e-5	1.86e-2	−0.87	2.13e-5	7.98e-3	−0.66
BORCS8	BLOC-1-related complex subunit 8	2.91e-5	1.86e-2	−0.86	1.61e-5	7.31e-3	−0.64
SLC25A24	Solute carrier family 25 member 24	5.87e-5	2.10e-2	−0.85	1.10e-5	6.82e-3	−0.71
MBOAT2	Membrane bound O-acyltransferase domain containing 2	1.73e-4	2.35e-2	−0.76	7.00e-6	6.82e-3	−0.84
ACOX2	Acyl-CoA oxidase 2	3.59e-5	1.86e-2	−0.74	2.78e-5	8.77e-3	−0.61
TNFRSF11B	TNF receptor superfamily member 11b	3.13e-5	1.86e-2	−0.74	3.02e-7	1.96e-3	−1.18
NA*	ENSG00000260912*	3.47e-3	4.91e-2	−0.74	4.04e-5	9.30e-3	−1.05
NA*	ENSG00000234119*	3.74e-5	1.91e-2	−0.71	8.20e-6	6.82e-3	−0.69
SNX24	Sorting nexin 24	2.46e-3	4.32e-2	−0.70	5.16e-5	9.83e-3	−0.86
ANP32 A	Acidic nuclear phosphoprotein 32 family member A	8.22e-5	2.26e-2	−0.70	2.15e-5	7.98e-3	−0.62
CANX	Calnexin	5.44e-4	2.89e-2	−0.70	2.32e-6	5.52e-3	−0.95
TMX2P1	Thioredoxin related transmembrane protein 2 pseudogene 1	2.87e-4	2.49e-2	−0.70	5.42e-5	9.96e-3	−0.63
B4GALT4	Beta-1,4-galactosyltransferase 4	8.17e-4	3.19e-2	−0.68	3.53e-6	6.82e-3	−1.17
SNX4	Sorting Nexin 4	1.09e-4	2.28e-2	−0.67	4.74e-5	9.65e-3	−0.61
TSPYL4	TSPY like 4	1.41e-4	2.35e-2	−0.66	8.55e-6	6.82e-3	−0.71
PHLDA2	Pleckstrin homology like domain family A member 2	1.67e-3	3.74e-2	−0.60	4.19e-5	9.37e-3	−0.61
TP53I3	Tumor protein p53 inducible protein 3	1.31e-4	2.35e-2	−0.59	2.61e-5	8.77e-3	−0.60
ARHGAP19	Rho GTPase activating protein 19	2.25e-4	2.37e-2	0.59	7.93e-6	6.82e-3	0.74
LAMC1	Laminin subunit gamma 1	6.03e-4	2.95e-2	0.60	6.01e-6	6.82e-3	0.96
STK17B	Serine/threonine kinase 17b	1.32e-3	3.54e-2	0.61	5.71e-5	1.00e-2	0.65
NRP2	Neuropilin 2	2.83e-3	4.55e-2	0.65	4.83e-5	9.65e-3	0.85
IDNK	IDNK, gluconokinase	2.18e-3	4.17e-2	0.67	2.16e-4	1.34e-2	0.62
KTN1	Kinetin 1	4.50e-5	2.05e-2	0.68	1.88e-5	7.93e-3	0.60
RACGAP1	Rac GTPase activating protein 1	2.77e-4	2.49e-2	0.72	6.11e-5	1.00e-2	0.65
CDK6	Cyclin dependent kinase 6	8.63e-5	2.26e-2	0.75	4.60e-6	6.82e-3	0.86
SPA17	Sperm autoantigenic protein 17	1.51e-4	2.35e-2	0.77	2.00e-5	7.98e-3	0.66
LINC00467	Long intergenic non-protein coding RNA 467	3.30e-5	1.86e-2	0.80	8.62e-6	6.82e-3	0.68
BUB1B	BUB1 mitotic checkpoint serine/threonine kinase B	3.99e-5	1.94e-2	0.83	1.84e-5	7.89e-3	0.70
BIK	BCL2 interacting killer	3.11e-5	1.86e-2	0.86	2.01e-6	5.18e-3	0.87
SH3D19	SH3 domain containing 19	1.22e-4	2.35e-2	0.89	1.05e-5	6.82e-3	0.83
EIF4EBP2	Eukaryotic translation initiation factor 4E protein binding 2	7.50e-5	2.26e-2	0.92	2.09e-5	7.98e-3	0.72
HMMR	Hyaluronan mediated motility receptor	4.07e-5	1.95e-2	0.95	1.17e-5	6.82e-3	0.74
KLHL15	Kelch like family member 15	3.55e-4	2.64e-2	0.95	2.46e-4	1.42e-2	0.59
DYRK2	Dual specificity tyrosine phosphorylation regulated kinase 2	4.09e-4	2.69e-2	0.97	3.68e-5	9.30e-3	0.80
ZNF268	Zink finger protein 268	3.95e-4	2.66e-2	0.98	8.72e-5	1.07e-2	0.67
NUP98	Nucleoporin 98	9.71e-6	1.86e-2	0.98	6.76e-6	6.82e-3	0.73
MTMR6	Myotubularin related protein 6	8.09e-4	3.19e-2	0.99	1.18e-4	1.20e-2	0.78
PTP4A1	Protein tyrosine phosphatase type IVA, member 1	5.08e-4	2.85e-2	1.04	1.94e-4	1.31e-2	0.80
GOLT1B	Golgi transport 1B	1.36e-5	1.86e-2	1.04	1.95e-5	7.98e-3	0.63
RPS27L	Ribosomal protein S27 like	6.91e-6	1.86e-2	1.05	1.06e-5	6.82e-3	0.67
MALAT1	Metastasis associated lung adenocarcinoma transcript 1	1.46e-3	3.66e-2	1.09	1.06e-5	6.82e-3	1.67
LAMP2	Lysosomal associated membrane protein 2	3.30e-4	2.62e-2	1.10	4.75e-5	9.65e-3	0.98
SYPL1	Synaptophysin like 1	5.69e-6	1.86e-2	1.11	5.63e-6	6.82e-3	0.73
ARRDC4	Arrestin domain containing 4	1.55e-3	3.70e-2	1.24	2.11e-3	3.15e-2	0.64
MBNL1	Muscleblind like splicing regulator 1	1.73e-6	1.86e-2	1.34	5.91e-6	6.82e-3	0.73

p-value, q-value and log_2_ fold change (FC) from the two differential mRNA expression analyses are shown for each gene ID. The Gene names are retrieved from Ensembl. The table is sorted on SW480 log_2_ FC. *Gene id and gene name are not available. The Ensembl id is provided as identifier. **FC = log_2_ fold change.

### Identification of the most significantly deregulated gene modules

In order to gain understanding from the sets of genes differentially expressed by *AURKA* knockdown, we applied the Heinz algorithm^26^. Heinz integrates significant deregulation (the p-values from the DEA described above) with molecular protein interaction data (PPI network data from STRING)^27^. The algorithm looks for significantly deregulated gene modules (sets of genes connected in the PPI network), which might include genes that show no expression changes, based on the intuition that some regulatory effects may involve changes that are not visible at the expression level. The genes in these modules can therefore be considered ‘functionally’ deregulated. First, weights are assigned to each node reflecting its p-value from the DEA. The chosen FDR cut-off determines which nodes are assigned positive weights (p-value below threshold) or negative (above threshold). The Heinz algorithm then computes the most significantly deregulated gene module, which is based both on the connectivity of the PPI network and the assigned weights. By thus combining the differential expression and protein interaction data, we can identify the most significantly deregulated gene modules (or functionally deregulated genes) upon *AURKA* downmodulation.

**Figure 2:**
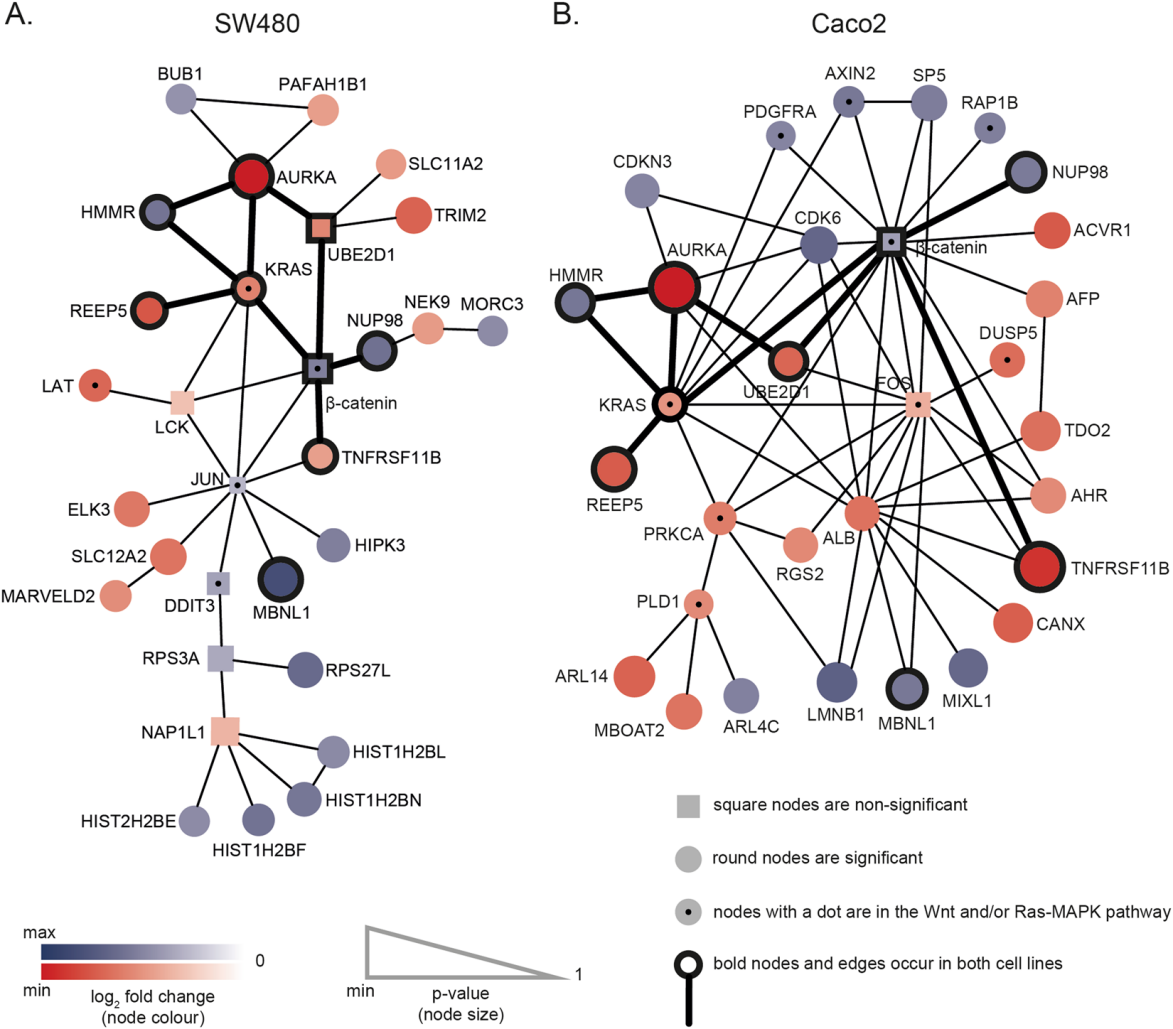
Significantly deregulated gene modules in SW480 (**A**) and Caco2 (**B**) derived from integrating p-values from differential mRNA expression analysis (DEA) upon downmodulation of *AURKA* and STRING protein-protein interaction network data using the Heinz algorithm. (**A**) and (**B**) Based on the chosen FDR threshold of 6.21e-4 for SW480 and 2.40e-4 for Caco2 in the Heinz analysis, round nodes are significant (p-value lower than the FDR threshold), and squared nodes are non-significant (p-value above the FDR threshold). The size of the nodes is based on the p-values from the DEA: a lower p-value corresponds to a bigger node. The colour of the nodes indicates the direction of the fold change with respect to *AURKA* downmodulation in the DEA. Red nodes are downregulated and blue nodes upregulated. The colour intensity of the nodes is correlated with the magnitude of the fold change, where the greatest intensities represent the highest absolute fold change. The nodes of the nine genes occurring in both modules are outlined in black and the direct edges between these are in bold. The nodes representing proteins in the Wnt or Ras-MAPK pathways have a black dot.

The FDR threshold was set to 6.21e-4 for SW480 and 2.40e-4 for Caco2. These thresholds were selected so that only the 50 most significantly deregulated genes were assigned positive weights. This selection allowed us to focus this analysis on gene modules of interpretable sizes (see Materials and Methods for further details). Figure 2 shows the most significantly deregulated gene modules for SW480 and Caco2. For SW480 the most significantly deregulated gene module consisted of 30 differentially expressed nodes (15 up and 15 down; see Fig. 2A and Supplementary Table S3). Twenty-three were significantly deregulated (positive weight, circles in Fig. 2A) and seven non-significant (negative weight, squares in Fig. 2A). For Caco2 the most significantly deregulated gene module consisted of 31 differentially expressed nodes (13 up and 18 down; see Fig. 2B and Supplementary Table S4). Twenty-nine were significantly deregulated (positive weight, circles in Fig. 2B) and two non-significant (negative weight, square in Fig. 2B. Note that remaining genes out of the 50 genes with positive weights do not occur in the largest connected module, because they are not connected in the PPI network. The intersection between the most significantly deregulated gene modules of each of the two cell lines contains nine genes: *AURKA*, *β-catenin*, *HMMR*, *KRAS*, *MBNL1*, *NUP98*, *REEP5*, *TNFRSF11B* and *UBE2D1* (black outline in nodes in Fig. 2A,B). These are all directly connected in the modules (bold edges in Fig. 2A,B), except for *MBNL1*, which is connected via one intermediate node: in SW480 via *JUN* to *KRAS*, *β-catenin* and *TNFRSF11B*, and in Caco2 via *ALB* to *KRAS*, *AURKA*, *β-catenin* and *TNFRSF11B*, and via *FOS* to *KRAS*, *UBE2D1*, *β-catenin* and *TNFRSF11B*.

This integrative approach, using the p-values from the DEA and the PPI network data from STRING determined 30 and 31 genes in the most significantly deregulated gene modules for SW480 and Caco2, respectively. This analysis identified three genes for SW480 (*JUN*, *RPS3A* and *LCK*) and two genes for Caco2 (*β-catenin* and *FOS*), which were not significantly deregulated in the DEA.

### Proteins and interactions involved in enhanced Wnt and Ras-MAPK signalling by AURKA

**Figure 3:**
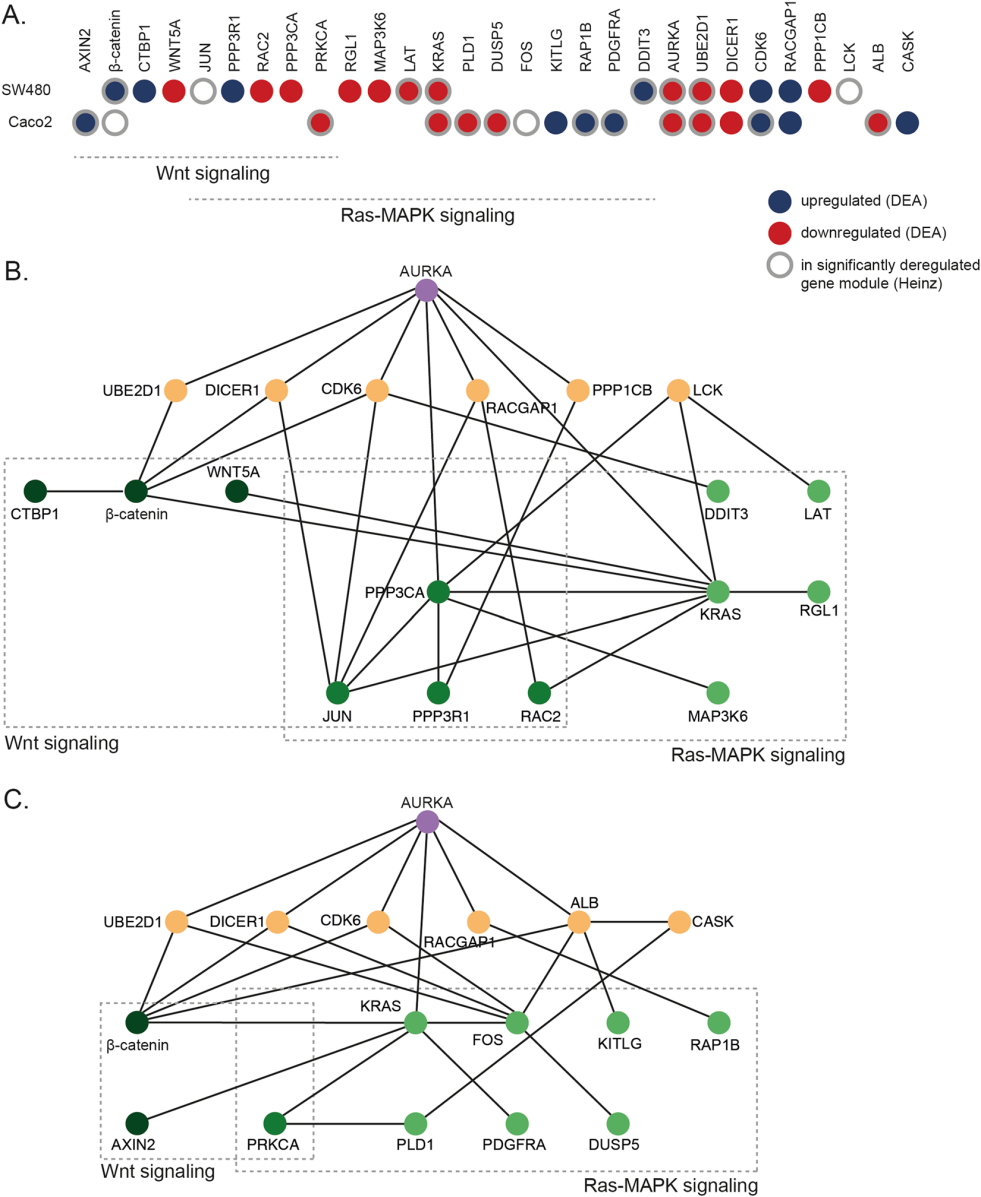
Wnt and Ras-MAPK pathway genes and interacting genes with AURKA, significantly deregulated (DEA) or in the most significantly deregulated gene module (Heinz) in the two cell lines, SW480 and Caco2. (**A**) Overview of AURKA, the genes in the Wnt and Ras-MAPK pathways, and the genes connecting these, either significantly deregulated (DEA; q-value < 0.05, red for down and blue for upregulation) or found in the most significantly deregulated gene module (Heinz, grey edges) in the two cell lines. (**B**) and (**C**) Interactions between AURKA (top-middle, purple) and the Wnt and Ras-MAPK signalling genes (green shades) deduced together with additional ‘connecting genes’ (orange) using a shortest path analysis in SW480 (**B**) and Caco2 (**C**).

We then set out to annotate Wnt and Ras-MAPK signalling genes that were either significantly deregulated in the gene-centric DEA or found in the most significantly deregulated gene modules in the integrative network-aware Heinz analysis. In total, 20 genes in either of these two signalling pathways were identified: four genes in the Wnt pathway (*AXIN2*, *β-catenin*, *CTBP1* and *WNT5A*), eleven in the Ras-MAPK pathway (*DDIT3*, *PDGFRA*, *RAP1B*, *KITLG*, *FOS*, *DUSP5*, *PLD1*, *KRAS*, *LAT*, *MAP3K6*, *RGL1*), and five in both pathways (*PRKCA*, *PPP3CA*, *RAC2*, *PPP3R1* and *JUN*) (see Fig. 3A). Two of these 20 deregulated genes, *KRAS* and *β-catenin*, appeared in both cell lines. The other 18 genes behaved cell line specific in these analyses: ten genes were found in SW480 only and eight genes were found only in Caco2 (Fig. 3A). When looking at Wnt and Ras-MAPK genes compared to all selected genes by the DEA (significantly deregulated) and the Heinz analysis (part of the most significantly deregulated gene module), respectively, we see different enrichments. For the DEA this was 11/292 (4%) and 8/154 (5%) for SW480 and Caco2, respectively (coloured nodes in Fig. 3A, and totals in Fig. 1C). On the other hand, for the Heinz analysis this was 5/30 (17%) and 9/31 (29%) for SW480 and Caco2, respectively (black dots vs. all nodes in Fig. 2A,B). Hence, Wnt and Ras-MAPK signalling genes were much more enriched in the deregulated modules selected by the network-aware Heinz analysis compared to the DEA, suggesting that their mutual interaction within the associated PPI network modules is important for CRC progression.

The most significantly deregulated gene modules also suggest interactions between AURKA and the Wnt and Ras-MAPK pathway (Fig. 2). Only KRAS is directly connected to AURKA, whereas the other genes are indirectly connected to AURKA via one or more genes. In order to identify interactions between AURKA and the remaining genes of the 20 Wnt and Ras-MAPK genes, we applied a shortest path analysis through the protein interaction network. This analysis was repeated for each of the cell lines, SW480 and Caco2. We used a subset of the STRING PPI only consisting of the genes that were either significantly deregulated in the DEA or part of the most significantly deregulated module from the Heinz analysis. The shortest path between each of these genes to AURKA was determined for SW480 (Fig. 3B) and Caco2 (Fig. 3C). Some of these interactions are directly from AURKA to Wnt and Ras-MAPK genes, but most are indirectly via additional interactions outside Wnt or Ras-MAPK signalling. For both cell lines there were six such external ‘connecting genes’: *LCK* and *PPP1CB* in SW480, and *ALB* and *CASK* in Caco2, and four shared: *UBE2D1*, *DICER1*, *CDK6* and *RACGAP1*. We propose that these genes could be important to explain the observed correlation between AURKA modulation and Wnt and Ras-MAPK activity.

### TCGA CRC data analysis

The Wnt and Ras-MAPK pathway proteins and their molecular interactions with AURKA identified in this study are based on two CRC cell lines with 20q gain where *AURKA* is downmodulated. These results provide us with a better understanding of possible interconnections between AURKA and Wnt and Ras-MAPK signalling, however, to gain insight into the CRC progression it is necessary to study these genes from a cellular context that better represents the physiological environment. In CRC tissue samples where epithelial neoplastic cells interact with their tumour microenvironment the biology is more complex and heterogeneous than in isolated epithelial cancer cell lines.

We analysed publicly available CRC samples from TCGA (RNA-seq and somatic copy number aberration data) to see if we could recover the significantly deregulated genes from the CRC cell line DEA. Out of 330 microsatellite stable tumour samples, 217 (65.76%) had *AURKA* copy number aberration, determined by a segment mean threshold of 0.4 in the somatic copy number aberration data (see Materials and Methods). DEA was performed between the *AURKA*-no-gain and *AURKA*-gain samples using the RNA-seq expression data. Note this atypical order of the sample groups (no-gain vs. gain instead of gain vs. no-gain) is to ensure proper comparison to the distinctive setup of the cell line experiments where *AURKA* is downmodulated (see Materials and Methods). *AURKA* was significantly deregulated (p-value = 1.62e-26), demonstrating the correlation between increased *AURKA* count and copy number gain. In total, 1308 genes were significantly deregulated in the *AURKA* no-gain samples with a significance threshold of the adjusted p-value less than 10^−5^, 751 up and 557 down (Supplementary Table S6). We then investigated the significance and directionality of the genes located on 20q. First, we retrieved the Ensembl ids for the genes located on 20q11-20q13.33 from BioMart. Second, we mapped these Ensembl ids to the Ensembl ids in the TCGA samples, which resulted in 316 genes. Out of these, 168 were significantly deregulated, 164 down and 4 up. Thus, 163 genes located on 20q11-20q13.33 are significantly deregulated in the same direction as AURKA (Supplementary Table S7).

We compared the significantly deregulated genes in the TCGA *AURKA* no-gain samples (n = 1308) to the sets of genes significantly deregulated in the SW480 (n = 292) and Caco2 (n = 154) DEA (described above). A minor overlap was found; only 24 genes were significantly deregulated in the TCGA and either of the cell lines (14 in SW480 and 13 in Caco2). Further, the majority of these genes were deregulated in opposite directions between the two different settings (see Supplementary Fig. S4).

## Discussion

In this study we identified 20 Wnt and Ras-MAPK signalling genes and eight additional connecting genes that suggest possible mechanisms of interaction between AURKA and Wnt and Ras-MAPK signalling in a CRC cell line setting. We performed mRNA expression analysis on *AURKA* knockdown in two CRC cell lines, SW480 and Caco2, and applied three main computational approaches to interpret this expression data. First, we applied straightforward gene-centric DEA determining significantly deregulated genes. Second, we applied the Heinz algorithm, an integrative network-aware analysis, using the expression data and the STRING PPI network data to determine significantly deregulated gene modules. Third, we applied a shortest path analysis determining connections between AURKA and Wnt and Ras-MAPK signalling genes that were significantly deregulated in the first two analyses, DEA and Heinz. The DEA resulted in the largest set of genes, however it does not by itself reveal how their deregulation may be intertwined. The Heinz integrative analysis clearly revealed in an unbiased way the involvement of the Wnt and Ras-MAPK pathways, as well as indicated some plausible mechanisms of interaction between AURKA and both pathways. Finally, the shortest path analysis added 8 connecting genes that may be relevant to explain how *AURKA* modulation may affect the activity of these pathways, and thereby further induce proliferation in developing carcinoma cells.

In previous studies interactions between AURKA and Wnt signalling have been observed in gastric cancer cell lines^18,19^ and glioma-initiating cells^20^, and interactions between AURKA and Ras-MAPK signalling have been observed in kidney cells^21^, pancreatic cancer cells^22^ and multiple myeloma^23^. Thus the link between AURKA and Wnt and Ras-MAPK signalling is not new in general, but the specific proteins that are deregulated differ between the different cellular contexts. For instance, GSK3B and AXIN1 are two proteins deregulated by AURKA leading to stimulation of Wnt signalling in gastric cancer cell lines^18,19^ and glioma-initiating cells^20^, respectively. Activation of Ras-MAPK by AURKA has been attributed to interaction between AURKA and the H-RAS/Raf-1 complex in kidney cells^21^. In our analysis, in the two CRC cell lines, SW480 and Caco2, differential expression of *GSK3B*, *AXIN1* and *HRAS*, was not recovered, however, *AXIN2* and *KRAS*, homologous of *AXIN1* and *HRAS*, respectively, were recovered. Besides the differences in the cellular contexts, a plausible explanation for not recovering *GSK3B* and *AXIN1* in our analysis could be that GSK3B phosphorylation and AXIN1 binding to AURKA and β-catenin involve changes at the protein level, which would not be visible in our mRNA expression data. Another limitation of our experimental setup is that *AURKA* is the only gene that is downmodulated, whereas in an *in vivo* setting more genes on 20q are deregulated together with *AURKA*. One example is *TPX2*, which just as with *AURKA* is located on 20q, and implied to be involved with gain of 20q^11^, however, in our analysis TPX2 is not modulated.

Based on our analysis, the genes in the Wnt and Ras-MAPK pathways affected by *AURKA* downmodulation were mostly unique per cell line. In the Wnt pathway, only *β-catenin* was affected in both cell lines, and in the Ras-MAPK pathway, it was only *KRAS*. It should be noted, however, that these genes are at the core of their respective pathways, which suggests that in both cellular contexts, perturbing these pathways may be very important. The molecular differences can be explained by the cause or the effect of the perturbations being dependent on the particular cellular context. On the other hand, the ‘connecting genes’ (connecting AURKA to the Wnt and Ras-MAPK signalling genes) were mostly similar between the cell lines (four out of six genes shared in both). While both SW480 and Caco2 are CRC cell lines with 20q gain, AURKA overexpression, and TP53 mutation, they also show differences. Firstly, the cell lines originate from different individuals, and therefore different germline variations. Secondly, the two cell lines have progressed to carcinomas independently. One specific difference is that *KRAS* is mutated in SW480 but not in Caco2^25^. However, their genetic differences are much more extensive than this (see DNA copy number profiles in Supplementary Fig. S1). Consequently, the underlying molecular biology of SW480 and Caco2 differs and they are therefore expected to respond differently to a perturbation such as *AURKA* downmodulation when looking at the expression of individual genes, although the overall changes in pathways could still be the same. For instance, KRAS is one of the most connected genes in both cell lines in our two network aware analyses (Figs 2 and 3), while at the same time the differentially expressed networks upon AURKA knockdown show different genes connected to KRAS in both cell lines. This could potentially be due to the different mutation status of KRAS between the cell lines. For instance, in the shortest path analysis (Fig. 3) KRAS is connected to AURKA and β-catenin in both cell lines. However, in SW480, where KRAS is mutated, KRAS in addition is connected to LCK, WNT5A, PPP3CA, JUN, and RAC2. Whereas, in Caco2, KRAS is in addition connected to FOS, AXIN2, and PRKCA.

The four consensus molecular subtypes (CMS) of colorectal cancer distinguish differences in signalling activities^28^. CMS2 and CMS4 are the most common chromosomal instable subtypes, both containing 20q gain, with CMS2 having a better prognosis than CMS4. CMS2 show increased proliferation, whereas CMS4 exhibit invasion. In a recent study^29^, they found that four genes were sufficient to predict CMS4: *PDGFRA*, *PDGFRB*, *PDGFC* and *KIT*. Interestingly, in the current study, *PDGFRA* (p-value = 1.28e-05) and the *KIT* ligand, *KITLG* (p-value = 3.19e-05), were significantly deregulated in Caco2, but not in SW480 (possibly explained by the different genetic backgrounds). Thus upon *AURKA* downmodulation Caco2 resembles the CMS4 subtype, and it can be implied that Caco2 also displays the CMS4 phenotype, invasion.

The cell line results presented in this paper suggest possible interconnections between AURKA and Wnt and Ras-MAPK signalling. Analysis of the CRC tissue samples from the TCGA data provided results from a more appropriate cellular context for studying CRC progression. There was an overlap of 24 genes when comparing the significantly deregulated genes from the TCGA analysis and the cell line analyses. However, most of these overlapping significantly deregulated genes were deregulated in opposite directions between the two different settings (Supplementary Fig. S4). We should here take into account the big differences between these two settings. First, in the tissue samples from TCGA *AURKA* is gained as a result of gain in 20q, on which many more genes than only *AURKA* are located and affected (Supplementary Table S7). In the cell line experiments, however, only *AURKA* is downmodulated, whereas all the other genes on 20q have remained unchanged. Second, in a tumor, epithelial neoplastic cells interact with their tumor microenvironment. Thus, the tissue samples are much more complex than the cell lines, and most likely there would be feedback mechanisms active in this environment that do not operate in the isolated cell lines. Third, a tumor is very heterogeneous, whereas a cell line is much more homogeneous. Thus, the presence of multiple clones within a tumor might also mask the effect of *AURKA* gain present in a minority of the clones in the tumor. Thus, we believe that because of these big differences between the cell line and tissue settings, a gene that is deregulated in both cases does not necessarily have to be deregulated in the same direction, but the overlap of deregulated genes, and their pathways, provides a good starting point for further investigation of the mechanisms between AURKA and Wnt and Ras-MAPK signalling. At the same time, this also underlines the importance of cellular context when studying regulation of cancer.

In summary, gene-centric and network-aware analysis of CRC cell lines with *AURKA* knockdown shows that 20 Wnt and Ras-MAPK signalling genes, occurring in connected PPI networks in two CRC cell lines, are significantly deregulated by AURKA. The responses in the Wnt and Ras-MAPK pathways were different in the two cell lines; only *KRAS* (Ras-MAPK) and *β-catenin* (Wnt) were deregulated in both. Further, in both cell lines four genes connected *AURKA* to the deregulated genes in the Wnt and Ras-MAPK pathways: *CDK6*, *UBE2D1*, *DICER1* and *RACGAP1*. These results suggest possible genes and mechanisms for the interplay between AURKA and Wnt and Ras-MAPK signalling that are at the same time generic and unique between different CRC settings. Further investigation of the importance of Wnt and Ras-MAPK enhanced signalling by AURKA in CRC and the role of these genes for these interactions, will lead to a better understanding of the molecular mechanisms underlying CRC progression.

## Materials and Methods

### Cell culture and transfection with small interfering RNA (siRNA)

SW480 cells were grown in Dulbecco’s modified Eagle’s medium (DMEM; Lonza, Verviers, Belgium) containing 10% fetal bovine serum (FBS) (HyClone; Perbio Science, Etten-Leur, The Netherlands), and Caco2 cells were grown in RPMI 1640 (Lonza) containing 20% FBS. Both cell culture media were supplemented with 2 mM L-glutamine, 100 IU/ml sodium penicillin (Astellas Pharma B.V., Leiderdorp, the Netherlands), and 100 mg/ml streptomycin (Fisiopharma, Palomonta (SA), Italy). Transfection with siRNA pools (SMARTpools) from Dharmacon (Lafayette, Colorado, USA) was performed 24 h after seeding according to the manufacturer’s recommendations. A final siRNA concentration of 30 nM was obtained using DharmaFECT3 reagent (1:1000 dilution) for both cell lines. A non-targeting control siRNA pool (Non-Targeting Pool 2; D-001206-14) was used as a negative control.

### RNA isolation

Total RNA was isolated using TriZol reagent (Invitrogen, Breda, the Netherlands) and subjected to purification using RNeasy Kit (Qiagen, Hilden, Germany). RNA concentrations and purities were measured on a Nanodrop ND-1000 spectrophotometer (Isogen, IJsselstein, the Netherlands). RNA quality was evaluated by generating an electropherogram on the Agilent Bioanalyzer 2100 using a RNA 6000 Nano-LabChip (Agilent Technologies, Santa Clara, CA, the Netherland). RNA integrity numbers (RIN) of >9.0 were considered as good quality RNA.

### Microarray expression analyses

Microarray expression experiments were performed on 4 × 44 K Agilent expression arrays (Agilent 60-mer SurePrint technologies), as described before^30^. Two biological replicates were performed for each siRNA experiment (both AURKA and non-targeting). Pre-processing and differential expression analysis (DEA) was done using the R-Bioconductor packages Limma^31^ and SVA-combat^32^. In short, conventional background correction was applied, followed by within-array normalization using Loess. Subsequently, batch-effect removal was applied for the replicate experiments, after which between-array normalization was done using the quantile method. The microarray data have been submitted to NCBI’s Gene Expression Omnibus (GEO) and are accessible through GEO Series accession number GSE108320. DEA between AURKA siRNA transfected cells and non-targeted siRNA control transfected cells was assessed by using linear modelling and empirical Bayes statistics. Finally, p-values were adjusted for multiple testing using conventional Benjamini Hochberg FDR correction. The threshold for significantly expressed genes was set at a q-value less than 0.05 and a fold change greater than 1.5 or less than −1.5. The significantly deregulated genes are available in Supplementary Table S1 for SW480 and Supplementary Table S2 for Caco2. GO enrichment analysis was performed using the ‘Gene Ontology enRIchment anaLysis and visuaLizAtion tool’ (http://cbl-gorilla.cs.technion.ac.il/).

### Probe ID mapping

The Agilent probe ids from the microarray expression experiments were mapped to their respective Ensembl ids and HGNC ids (when available) using BioMart mappings (‘Agilent WholeGenome 4 × 44 k v1 probe’) downloaded from www.ensembl.org/biomart/martview/ (January 2017). Additional mapping of HGNC ids to Ensembl ids was done using mappings from the Agilent website http://www.chem.agilent.com/cag/bsp/gene_lists.asp (Human Genome, Whole - Four-Plex, 44 K) (January 2017). We manually checked and mapped the significantly expressed genes for each cell line. Out of the 30,889 and 30,105 Agilent probe ids 8,270 and 8,149 could not be mapped to Ensembl ids for Caco2 and SW480, respectively. Where the same Ensembl id was mapped to multiple Agilent probe ids, the Agilent probe id with the lowest p-value was chosen. This filtering step resulted in 24,589 and 24,088 probes whereof 16,319 and 15,939 has unique Ensembl ids for Caco2 and SW480, respectively.

### Protein-protein interaction network

The *Homo sapiens* protein-protein interaction (PPI) network used in this analysis was retrieved from the STRING database (medium confidence: 0.400)^27^ (downloaded: January 2017). The proteins were mapped to Ensembl ids using BioMart mappings (as described in the previous section). Self- and duplicate interactions were removed. The network consisted of 680,790 PPIs.

To describe the nodes (genes) and edges (interactions), one node-file and one edge-file were constructed for each cell line, SW480 and Caco2, based on the intersection of nodes (Ensembl ids) in the *AURKA* knockdown DEA data and the STRING PPI data. The edges-files consisted of two columns with Ensembl ids representing the two interacting proteins. The node-file consisted of three columns: nodes (Ensembl id), p-values, and log_2_ fold change. The Caco2 edge-file consisted of 456,552 edges, and the SW480 edge-file consisted of 437,865 edges. The Caco2 node-file consisted of 12,249 nodes, while that for SW480 consisted of 12,082 nodes.

### Identification of the most significantly deregulated gene modules

The Heinz algorithm^26^ was applied to determine the most significantly deregulated gene module (sets of genes connected in the PPI network) for each cell line, SW480 and Caco2, using the constructed edge- and node-files. Each node in the network was assigned a weight reflecting its p-value from the DEA. A beta-uniform mixture model was fitted to the p-value distribution determining its parameters: the shape parameter, α, and the mixture parameter, λ^33^. The α parameter was 0.342 for SW480 and 0.329 for Caco2. Due to the high amount of low p-values, the λ parameter was approximated 0.0 in both cell lines. Before calculating the weights of the nodes we set the λ parameters to 0.1, which still gave a very reasonable fit. The Heinz algorithm then assigned weights to the nodes using the λ and α parameters, the p-values and a false-discovery rate (FDR) threshold. Positive weights were given to nodes with a p-value lower than the FDR threshold, whereas negative weights were given to nodes with a p-value greater than the FDR threshold. The FDR threshold for SW480 was 6.21e-4 corresponding to a p-value of 4.46e-5. The FDR threshold for Caco2 was 2.40e-4 corresponding to a p-value of 1.37e-5. The Heinz algorithm subsequently calculated the maximum-scoring subnetwork of the STRING PPI network that represents the most significantly deregulated gene module. The genes in these modules and their p-values and log_2_ fold changes are available in Supplementary Table S3 for SW480 and Supplementary Table S4 for Caco2.

Since the FDR threshold has an influence on how many nodes are assigned positive weights, it also has an influence on the size of the significantly deregulated gene module. We selected the FDR thresholds so that 50 genes had a positive weight for each cell line. These thresholds are a conservative choice to generate focus on the part of the network that shows the strongest deregulation. This means that genes with p-values just above the threshold may still be significantly deregulated. The resulting modules selected by Heinz are not very sensitive to the FDR setting, simply said the modules just incrementally grow or shrink, as the threshold is adapted.

### Determining Wnt and Ras-MAPK signalling genes

To define which genes are in the Wnt and Ras-MAPK pathways we downloaded the gene lists of the ‘Wnt signalling pathway’, ‘MAPK signalling pathway’ and ‘Ras signalling pathway’ from the KEGG database (January 2017)^34^. These genes were used to annotate Wnt and Ras-MAPK signalling genes in the results.

### Shortest path analysis

To find possible connections between AURKA and the signalling pathways of interest, we performed a shortest-path analysis. The shortest path is defined as the minimum number of edges required to travel from one node in the PPI graph to another. First, we reduced the STRING PPI network to a subnetwork only consisting of the significantly deregulated genes determined by the DEA and the genes located in the most significantly deregulated gene module in the Heinz analysis. We then applied the Python package NetworkX to compute all the shortest paths between AURKA and the Wnt and Ras-MAPK signalling genes, where the edges were unweighted.

### TCGA CRC data analysis

To compare the effect of AURKA on Wnt and Ras-MAPK signalling in CRC tissue data to our CRC cell line results we analysed the TCGA COADREAD^5^ RNA-seq and somatic copy number aberration data for 330 microsatellite stable tumour samples retrieved from Firehose (March 2017). First, the samples were labelled by *AURKA*-gain and *AURKA*-no-gain based on the copy number aberration data in the genome region 54,944,445-54,967,393 (genome build 19) encoding the *AURKA* gene. Samples with a segment mean greater than or equal to 0.4 were labelled *AURKA*-gain, whereas samples with a segment mean less than 0.4 were labelled *AURKA*-no-gain (Supplementary Table S5). The segment mean is the average of the tumour versus normal intensity log_2_ ratio, thereby describing the copy number aberrations. We selected the threshold of 0.4 following the assumption that the gain of *AURKA* also increases its expression (Supplementary Fig. S2). Second, DEA was performed between the *AURKA*-no-gain and *AURKA*-gain samples. The atypical choice of performing a no-gain vs. gain comparison (instead of gain vs. no-gain) is because of the distinctive setup of the cell line experiments themselves, where the non-perturbed cell lines have *AURKA* gain and the perturbed cell lines have an *AURKA* downmodulation (no-gain). Significantly expressed genes based on the DEA were determined at an adjusted p-value less than 10^−5^ (Supplementary Fig. S3). The significantly deregulated genes are available in Supplementary Table S6. We then investigated the significance and directionality of the genes located on 20q. First, we retrieved the Ensembl ids for the genes located on 20q11-20q13.33 from BioMart. Second, we mapped these Ensembl ids to the Ensembl ids in the TCGA samples. The TCGA DEA result for these genes is available in Supplementary Table S7. Finally, we determined the overlap of significantly deregulated genes between the TCGA DEA and the DEA of SW480 and Caco2 (Supplementary Fig. S4).

## Electronic supplementary material

Supplementary Figures

Supplementary Table S1

Supplementary Table S2

Supplementary Table S3

Supplementary Table S4

Supplementary Table S5

Supplementary Table S6

Supplementary Table S7
