# Supplementary material for: Aurora kinase A (AURKA) interaction with Wnt and Ras-MAPK signalling pathways in colorectal cancer
Source: Sci Rep. 2018 May 14;8:7522. doi: 10.1038/s41598-018-24982-z (PMC5951826; doi:10.1038/s41598-018-24982-z)
Supplement: Supplementary file 1 — Supplementary Figures [file 41598_2018_24982_MOESM1_ESM.pdf]

Supplementary information for

Aurora kinase A (AURKA) interaction with Wnt and Ras-MAPK signalling pathways in colorectal cancer

Annika Jacobsen[1], Linda J.W. Bosch[2], Sanne R. Martens-de Kemp[2], Beatriz Carvalho[2], Anke H. Sillars-Hardebol[3], Richard J. Dobson[4], Emanuele de Rinaldis[5], Gerrit A. Meijer[2], Sanne Abeln[1], Jaap Heringa[1], Remond J.A. Fijneman[2], K. Anton Feenstra[1]

Affiliations:
[1]Centre for Integrative Bioinformatics (IBIVU), Vrije Universiteit Amsterdam, Amsterdam, The Netherlands. [2]Department of Pathology, The Netherlands Cancer Institute, Amsterdam, The Netherlands. [3]Department of Pathology, VU University Medical Center, Amsterdam, The Netherlands. [4]Bioinformatics group at the NIHR Biomedical Research Centre for Mental Health (IOP) and the South London and Maudsley NHS Trust, London, UK. [5]The BRC Translational Bioinformatics Unit at the NIHR Biomedical Research Centre at Guy's and St Thomas' NHS Foundation Trust and King's College London, London, UK.

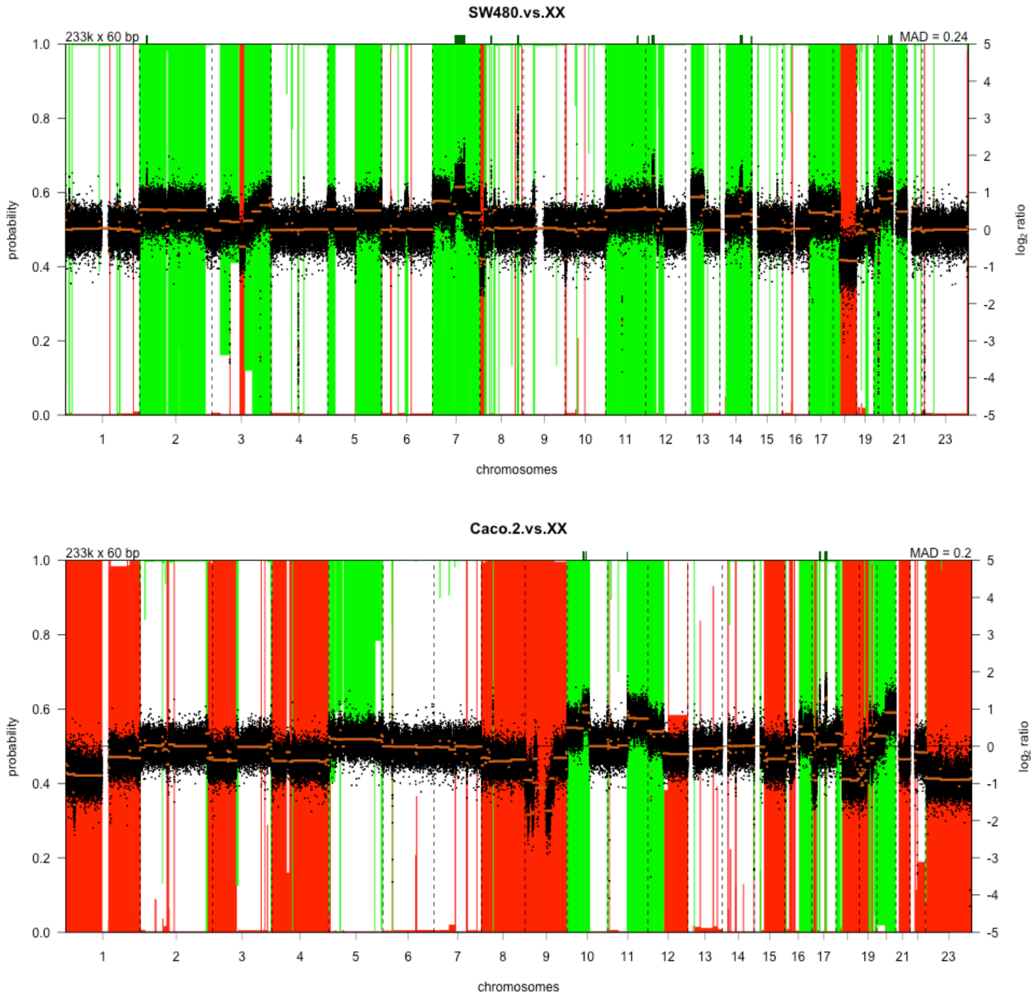

Supplementary Fig. S1. DNA copy number profile (aCGH) for SW480 (upper panel) and Caco2 (lower panel), respectively. Green represents regions with gain and red represents regions with loss (also represented by the positive/negative log_2 ratio). The figures show that 20q is gained in both cells lines, SW480 otherwise has many gained regions, whereas Caco2 has many lost regions.

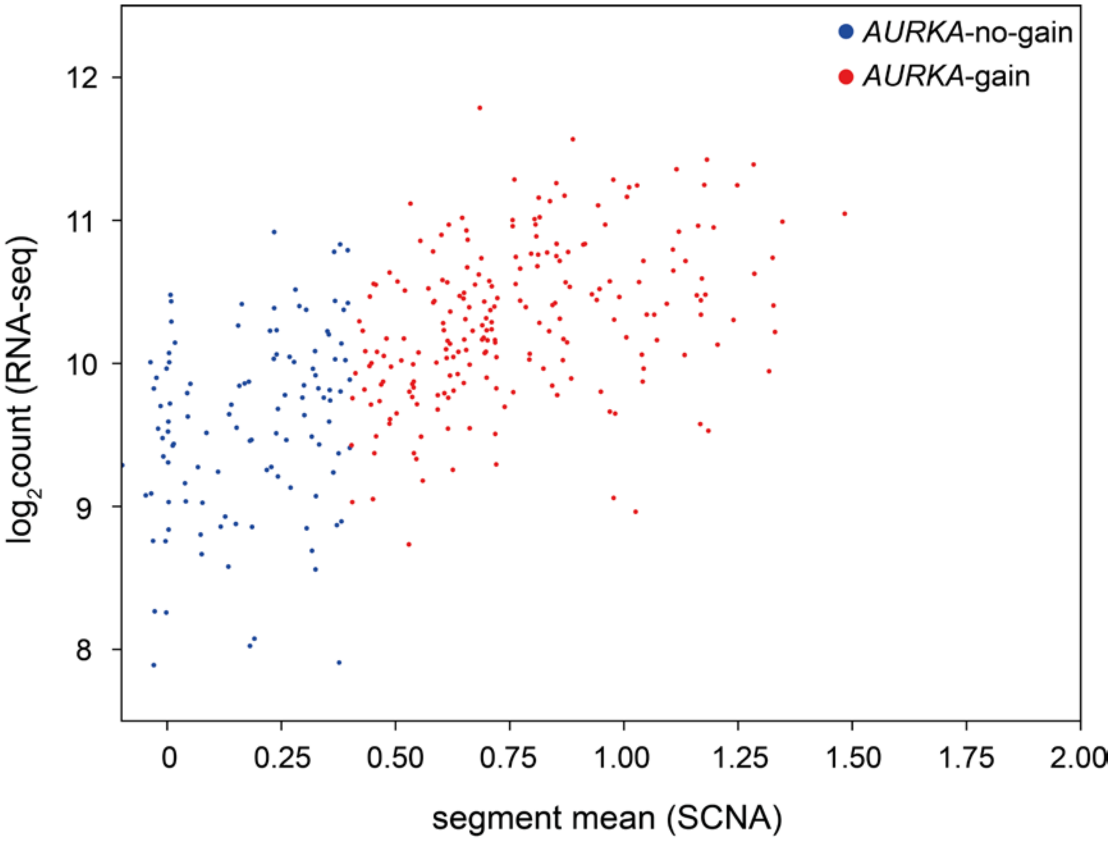

Supplementary Fig. S2. Segment mean (SCNA; DNA copy number) plotted against log_2count (RNA-seq, mRNA expression) for 330 *AURKA*-no-gain and *AURKA*-gain TCGA COADREAD samples. *AURKA*-gain samples were determined at a segment mean >= 0.4.

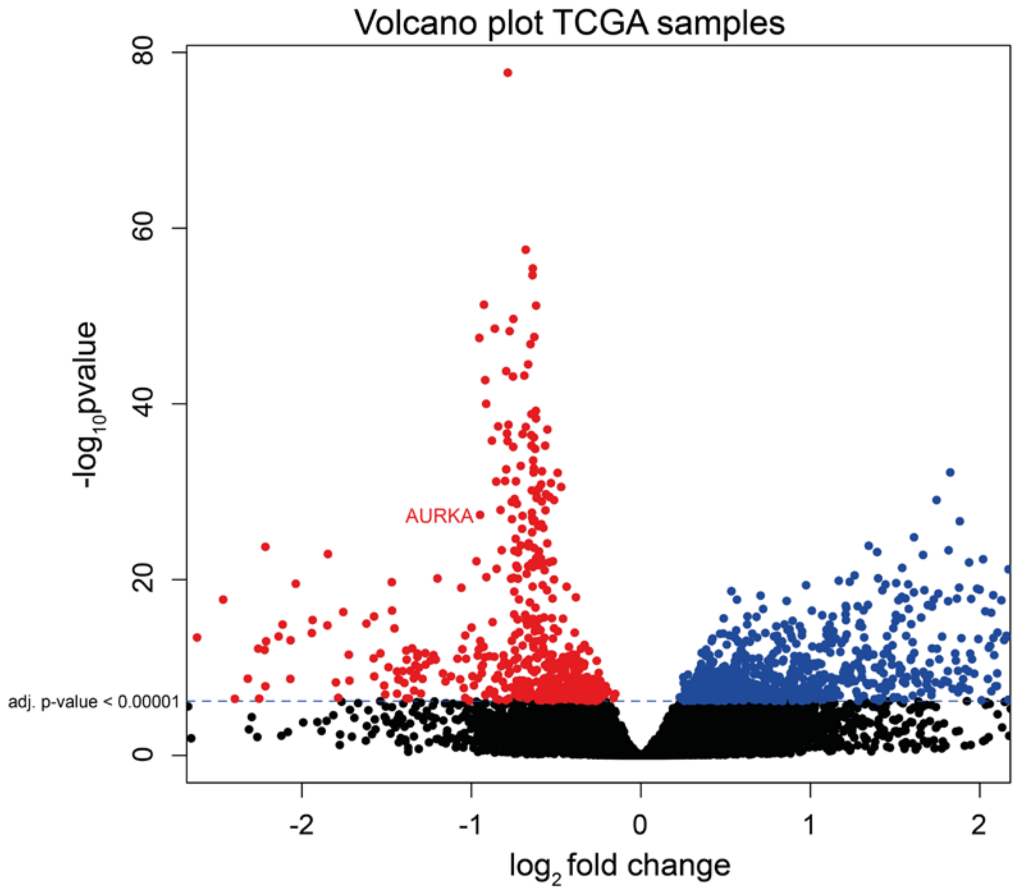

Supplementary Fig. S3. Volcano plot of the genes differentially expressed between 330 *AURKA*-no-gain and *AURKA*-gain TCGA COADREAD samples. The threshold for significantly deregulated genes was set at an adjusted p-value less than 10^{-5}.

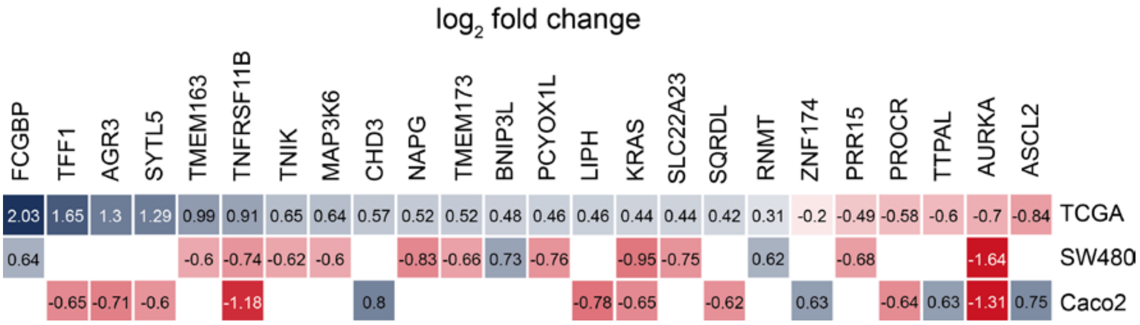

Supplementary Fig. S4. The significantly deregulated genes from the TCGA COADREAD DEA (*AURKA*-no-gain vs. *AURKA*-gain) compared to the sets of genes significantly deregulated in the DEA of the two cell lines, SW480 and Caco2, upon *AURKA* downmodulation. $\log_2$ fold change is shown for each gene in the intersection, further illustrated by blue for upregulation and red for downregulation.
